# The evaluation of antibiotic prescribing in general practice using a health insurance company database: penicillins use

**DOI:** 10.3325/cmj.2012.53.505

**Published:** 2012-10

**Authors:** Rachel Pipalova, Jiri Vlcek, Petra Matoulkova

**Affiliations:** Faculty of Pharmacy, Department of Social and Clinical Pharmacy, Hradec Kralove, Czech Republic

General practitioners often prescribe antibiotics for diagnoses that do not require antibiotic treatment, partially because of the fear of treatment failure (and alibism). They are also strongly influenced by patients’ requests, and the empirical use of antibiotics is the main way of infection treatment in general practice in the Czech Republic. All this leads to non-adherence to the clinical recommendations for infections treatment. However, the regular monitoring of total antibiotic consumption does not reveal the critical areas of antibiotic prescription. Therefore, we used the database of a health insurance company to evaluate the antibiotic prescribing habits of general practitioners in an age-stratified population. In this short text, we report our findings on the prescribing of penicillins (Anatomical Therapeutic Chemical group J01C) as an example.

The methodology based on the Anatomical Therapeutic Chemical/Defied Daily Doses system (ATC/DDD) has been used to survey antibiotic consumption worldwide and it allows making of international comparisons. However, it remains questionable whether this methodology is suitable to evaluate antibiotic prescribing in children and a more appropriate methodology should be developed (1). Researchers overcome this challenge by using the overall prevalence or prescribing rate, or they express the contact- or disease-based prescribing rate (2). Also in adult population, the assigned DDD may correspond to the most frequently prescribed dose. This may contribute to, eg, a higher consumption rate of co-amoxiclav. The need for higher doses of antibiotics to kill the intermediately sensitive pathogens increases this inaccuracy in DDD determination as well. We solved the problem of DDD determination using the number of prescriptions (corresponding to the number of treatment courses) for the calculations.

In our analysis from 2006-2010, we revealed considerable differences in narrow spectrum penicillins prescribing for different age groups. With advancing age, there was a dramatic decline in their overall consumption, which represented only about 5% of the total antibiotic prescribing for patients over 70 years of age. The opposite results were found in teenagers, where the consumption of narrow spectrum penicillins comprised almost 30% of the total antimicrobial consumption. This higher consumption rate may be partially explained by a higher incidence of streptococcal infections. An overall decreasing consumption of narrow-spectrum penicillins may be in part explained by the reluctance of patients to use penicillin because they believe that it is an obsolete and ineffective drug or the fear of allergy (past negative experience).

On the other hand, the use of broad-spectrum penicillins, namely co-amoxiclav, got higher in all age groups, including children. The use of aminopenicillins combined with beta-lactamase inhibitor in children younger than one year was four times higher than the use of aminopenicilins. It remains to be established whether higher consumption rate contributes to the high prevalence of resistant bacterial strains carriage in children (3).

Despite the fact that the total antimicrobial consumption in last few years has been relatively stable (although high), we observed striking variations in antibiotic prescribing in different age categories, confirming different but often stereotypical approaches of pediatricians and GPs to the treatment of infections and lack of adherence to the clinical guidelines.

Although the database of an insurance company provides only limited medical information, it enables the evaluation of the antibiotic use concerning the type of prescriber and population characteristics, which has not been routinely performed in the Czech Republic so far.
